# Controllable high-throughput high-quality femtosecond laser-enhanced chemical etching by temporal pulse shaping based on electron density control

**DOI:** 10.1038/srep13202

**Published:** 2015-08-26

**Authors:** Mengjiao Zhao, Jie Hu, Lan Jiang, Kaihu Zhang, Pengjun Liu, Yongfeng Lu

**Affiliations:** 1 Laser Micro/Nano Fabrication Laboratory, School of Mechanical Engineering, Beijing Institute of Technology, Beijing, 100081, China; 2 Department of Electrical Engineering, University of Nebraska-Lincoln, Lincoln, NE 68588-0511, USA

## Abstract

We developed an efficient fabrication method of high-quality concave microarrays on fused silica substrates based on temporal shaping of femtosecond (fs) laser pulses. This method involves exposures of fs laser pulse trains followed by a wet etching process. Compared with conventional single pulses with the same processing parameters, the temporally shaped fs pulses can enhance the etch rate by a factor of 37 times with better controllability and higher quality. Moreover, we demonstrated the flexibility of the proposed method in tuning the profile of the concave microarray structures by changing the laser pulse delay, laser fluence, and pulse energy distribution ratio. Micro-Raman spectroscopy was conducted to elucidate the stronger modification induced by the fs laser pulse trains in comparison with the single pulses. Our calculations show that the controllability is due to the effective control of localized transient free electron densities by temporally shaping the fs pulses.

Fused silica glass is widely used in microfluidic devices for bioassays, microreactors, and chemical/biological sensing. Conventional approaches to the fabrication of fused silica glass include hot embossing^1^, thermal reflow^2^,^3^, gray-tone (GT) photolithography^4^, and femtosecond (fs) laser direct writing (LDW)^5^. Most of the aforementioned methods require expensive photomasks. Because maskless processes, such as LDW, are usually inefficient and complex, they are not appropriate for large-area fabrication of microstructures on glasses.

So far, the technique of fs laser irradiation followed by chemical etching (FLICE) has attracted much attention. The process consists mainly of two steps: (1) permanent sample modification of the fused silica by focused fs laser pulses and (2) etching of the laser-modified zones by a hydrofluoric acid (HF) solution. Both planar and three-dimensional structures have been fabricated using FLICE^6^,^7^. However, a great challenge remains: how to improve the etching selectivity or etch rate? A fast and highly selective etching of the microchannels in fused silica substrates has been achieved using gaseous fluorine and hydrogen fluoride^8^. However, the experimental setup using the gaseous etching is complex and expensive.

In this study, we proposed a simple, efficient method for high-quality fabrication of microarrays on fused silica. A significant enhancement of etch rate was observed in the laser-modified zones by temporally shaping fs laser pulses. The fabrication can be flexibly controlled by adjusting the processing parameters, e.g., pulse delay, laser fluence, and pulse energy distribution ratio. In addition, micro-Raman spectroscopy was conducted to characterize the structural change in the irradiated regions. The mechanism of the etch rate enhancement and high controllability can be explained by the control of the localized transient free electron density and the corresponding change in photon absorption efficiency.

## Results

**Figure 1:**
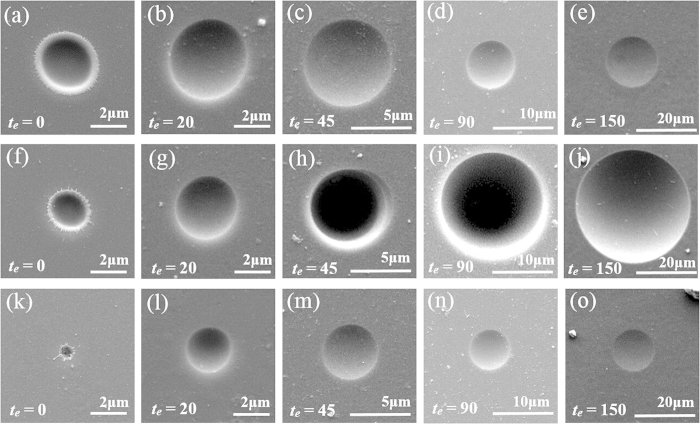
Morphology evolution of the sample surface exposed by (**a–e**) fs single pulse at a fluence of 9.46 J/cm^2^, (**f**–**j**) fs double pulses at a fluence of 9.46 J/cm^2^, pulse delay of 350 fs, and energy distribution ratio of 1:1, (**k**–**o**) fs double pulses at a fluence of 5.30 J/cm^2^, pulse delay of 350 fs, and energy distribution ratio of 1:1, at different stages of the etching process, where *t*_e_ represents the chemical etch time (min). The SEM images have varying scale bars.

In our study, single pulses and double pulses have been investigated and compared. All experimental results were obtained under the single-shot mode. Figure 1 shows the scanning eletron microscopy (SEM) images with varying scale bars for the morphology evolution on sample surfaces during the etching process, where *t*_*e*_ stands for the chemical etch time (min). For the samples exposed by a conventional single fs laser pulse of 9.46 J/cm^2^ (see Fig. 1(a–e)), before being treated with a HF solution, an elliptical crater with significant recast on the outer rim can be observed, as depicted in Fig. 1(a). As time goes on, the recast was smoothed out by the chemical etching process. The roughness was constantly reduced. A fine circularlyshaped concave structure was formed, as shown in Fig. 1(b–e). For the sample exposed by a fs laser pulse train (double pulses per train), the total fluence of which is also 9.46 J/cm^2^, the extension rate of the diameter is close to that of the single pulse at the early stage; but an obvious contrast change can be observed in the SEM image at *t*_*e*_ = 45 min, as shown in Fig. 1(f–h). Nevertheless, a drastic increase in the diameter took place at a late stage, which means that the pulse train increasingly reveals its effect in etch rate enhancement compared with a single pulse (see Fig. 1(d–e,i,j)).

**Figure 2:**
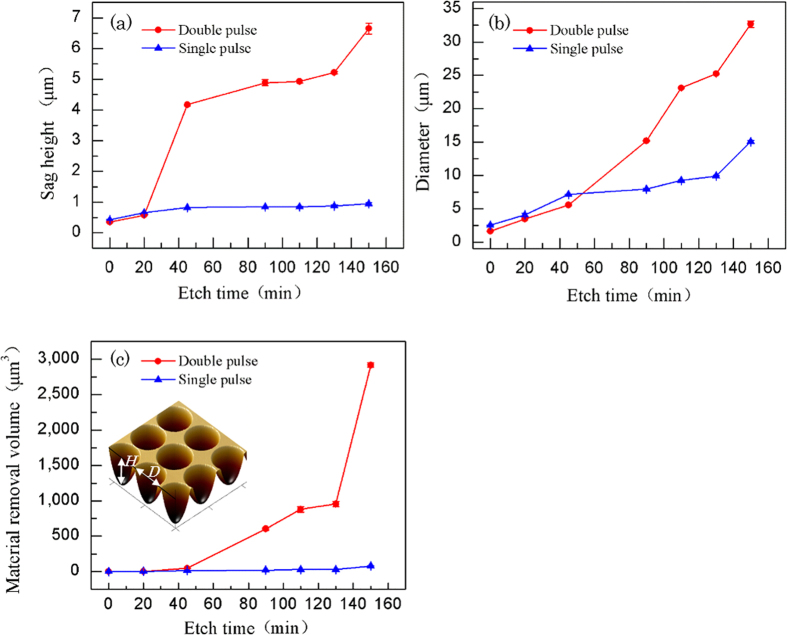
The plot of (**a**) the sag height, *H*, (**b**) the diameter, *D*, and (**c**) the material removal volume, *V*, versus the etch time, *t*_*e*_, at a laser fluence of 9.46 J/cm^2^ (the delay of the double pulse is 350 fs; the energy distribution ratio is 1:1).

To obtain more precise results, an atomic force microscope (AFM) was used to quantitatively characterize the evolution of the sag height (*H*) and the diameter (*D*) of the microstructures (Fig. 2(a,b)). Thus, the material removal volume (*V*) can be calculated by integrating the AFM profile data (Fig. 2(c)). Each data point represents an average of ten experimental data from different craters with error bars indicated. Initially, the fs laser pulse train induces smaller ablation than the conventional single pulse both in diameter and in sag height, which is in agreement with the experimental observations published^9^. However, after 20 min of etching, an abrupt change occurs in the sag height, which means that the double pulse induces a higher etch rate along the depth direction than the single pulse, as shown in Fig. 2(a). From Fig. 2(b), it can be clearly observed that a transition occurs at *t*_*e*_ = 45 min, when the diameter of the craters irradiated by a fs laser double pulse obviously exceeds the diameter of those irradiated by a single fs laser pulse. The material removal volume is a function of both the diameter and the sag height of the craters. From Fig. 2(c), a turning point of the material removal volume curve can be found at *t*_*e*_ = 45 min. Although the single pulse induces larger initial craters than the double pulse before chemical etching, the etch rate of the zones irradiated by a double pulse is much greater than that of a single pulse, which causes the double-pulse material removal volume to surpass the single pulse drastically along with the time. Here, we define the etch rate as the average volume of material removal per hour. From our calculations, we can obtain that at *t*_*e*_ = 150 min, the etch rate of the fs laser pulse train is about 37 times greater than that of the conventional single pulse.

**Figure 3:**
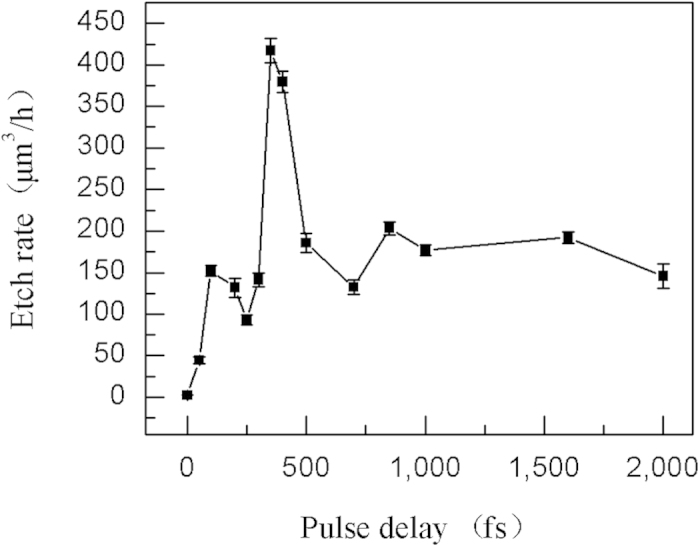
The plot of the etch rate versus the pulse delay at a laser fluence of 9.46 J/cm^2^ and pulse energy distribution ratio of 1:1 (the etch time is 90 min).

To quantitatively explore the morphology control of the microarrays, several processing parameters have been investigated. Figure 3 shows the dependence of the microstructure profiles on the double pulse delay ranging from 0 fs to 2 ps, with the irradiation fluence fixed at 9.46 J/cm^2^. The etch rate is obtained by averaging ten circularly shaped pits after the etching process in the ~8% HF solution for 90 min. For the pulse delays from 0 to 350 fs, the etch rate increases with fluctuation and reaches the maximum at 350 fs. Then an overall declining trend is observed with the increase in the pulse delay. 350 fs is the optimum pulse delay for the etch rate enhancement by the fs double pulse.

**Figure 4:**
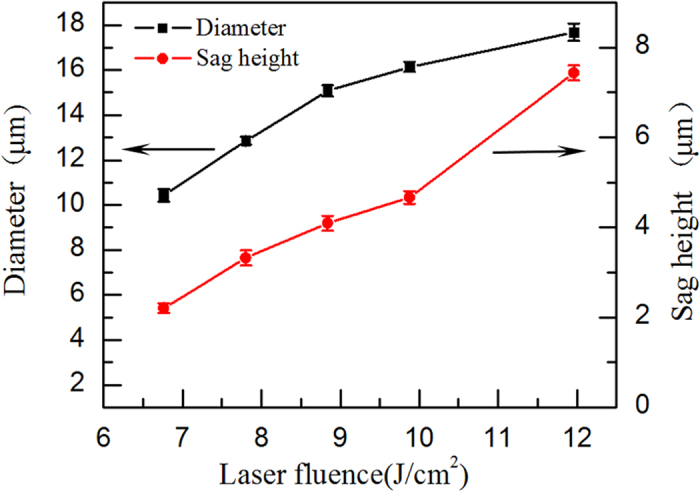
The plot of the diameter, *D*, and the sag height, *H*, versus the laser fluence at a pulse delay of 350 fs and a pulse energy distribution ratio of 1:1 (the etch time is 90 min).

The profile of the microstructures can also be controlled by the pulse energy. In Fig. 4, the pulse delay is fixed at 350 fs with the same energies for both subpulses. The sag heights and diameters of ten craters were averaged after a 90-min etching treatment. Both diameter and depth become higher as the pulse energy increases, due to extension of the laser-modified zones with the increasing laser pulse energy^10^. Based on this relationship, smaller structures can be fabricated when the laser fluence of the fs double pulse is reduced, as shown in Fig. 1(k–o). This implies that a temporally shaped pulse train has the potential to flexibly control the crater shapes.

**Figure 5:**
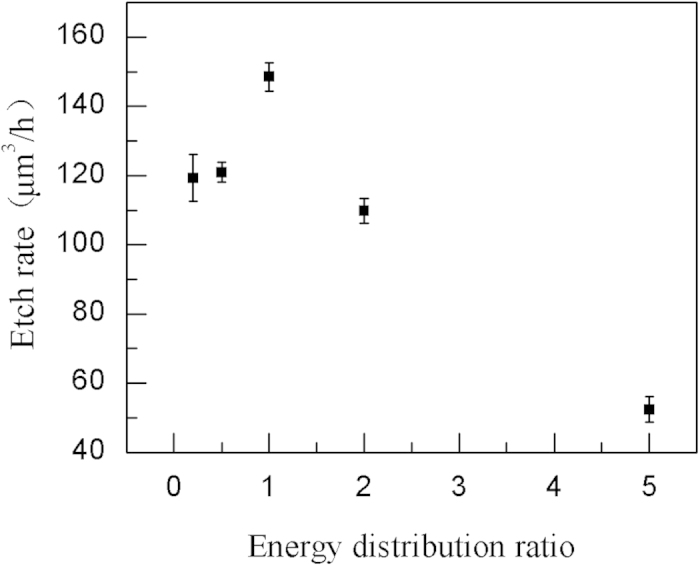
The relationship between the etch rate and the pulse energy distribution ratio. The total fluence is fixed at 9.46 J/cm^2^, the pulse delay is 350 fs and the etch time is 90 min.

Figure 5 shows the etch rate versus the pulse energy distribution ratio between the two subpulses. The pulse delay is fixed at 350 fs with a total fluence of 9.46 J/cm^2^. Each data point is an average of values from ten different pits. As shown in Fig. 5, the etch rate reaches the peak value at an energy ratio of 1:1 followed by a decrease. When the fluences of the two subpulses are equal, the highest etch rate is achieved. Therefore, the energy ratio of 1:1 is another optimum parameter in the fs laser pulse train enhanced chemical etching.

## Discussion

In our experiments, there are two points worth noting. One is that fs pulse train cause smaller ablation than the conventional single pulse. It is widely assumed that ablation takes place when the electron density reaches the critical density. During the ablation of fused silica by a fs laser, the peak free electron densities generated by the double pulse are less than those generated by a single pulse at the same total fluence, leading to smaller ablated structures. The former observation, which has been reported extensively, can be verified by the theoretical model described in our previous work^11^,^12^. Another phenomenon is that the chemical etch rate enhancement induced by fs laser pulse train is much greater when compared to single pulse. We could interpret this phenomenon using laser-induced structural changes in fused silica and the mechanisms of chemical etching in HF. The virgin atomic-scale structures of fused silica can be described as a broad random network, included in which are varisized rings consisting of repeated Si–O bonds. The number of the Si–O bonds in the ring structure widely ranges from 3 to 9^13^. Among them, the most stable and predominant structure is a regular 6-membered ring of quartz. When a fs laser beam is tightly focused into fused silica, the population of 3- and 4-membered ring structures in the irradiated regions increases at the expense of other types of rings^14^. The bridging bond angle is obviously reduced by the compressive stress created in the irradiated region, resulting in densification of the material. Silica is known to be attacked by aqueous HF through the following reaction scheme:


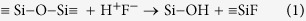


It has been proposed by Marcinkevicius and co-workers^15^ that the decrease in bridging bond angle in densified silica increases the reactivity of the oxygen atoms due to the deformed configuration of their valence electrons. The increase in reactivity is mainly responsible for a greater susceptibility to the HF etching in the modified zones with respect to the unmodified zones.

**Figure 6:**
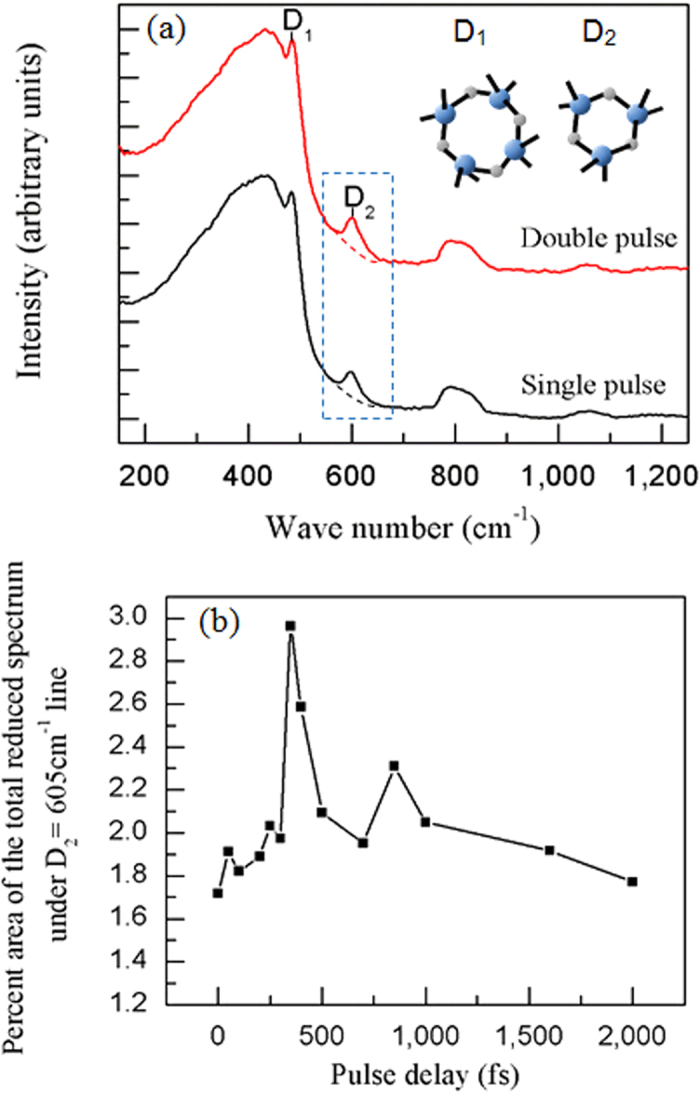
(**a**)The normalized Raman spectra of modified regions irradiated by fs laser single and double pulses in fused silica. The pulse delay of the double pulses is 350 fs. Dashed lines below the D_2_ peaks are baselines used in the peak area measurement in (**b**). The inset is the schematic diagram of 4- and 3-membered ring structures. (**b**) The percent area of the total reduced Raman spectrum under the D_2_ line versus different pulse delays. The total fluence is fixed at 9.46 J/cm^2^ and each site is irradiated by 500 shots. The measurements are conducted in a regions located 5 μm below the surface.

To verify the aforementioned discussion, micro-Raman spectroscopy was conducted to characterize the internal structure of the sample. Figure 6(a) shows the Raman spectra of fs-laser-modified zones in fused silica, where single and double pulses were investigated as comparison. The total fluence is fixed at 9.46 J/cm^2^ and the pulse delay of the double pulses is 350 fs. Each site is irradiated by 500 shots. The Raman spectra typically consist of a number of bands that can be assigned to the vibrations of different types of bonds in the glass network. We focus on the two peaks at 495 and 606 cm^−1^, which are often referred to as D_1_ and D_2_ defect lines. Pasquarello and Car^16^ assigned them to the 4- and 3-membered rings in the glass network, respectively. Since the D_1_ peak overlaps the main broad band at 445 cm^−1^, we choose D_2_ peak to quantify our results. The intensity of D_2_ is measured by drawing a baseline, the dashed spline curves shown in Fig. 6(a). The area between the Raman spectrum and the baseline is calculated and normalized against the peak height of the main line at 445 cm^−1^. According to calculation results, the percent area of the total reduced spectrum under D_2_ line of areas processed by fs laser single and double pulses are 1.7171 and 2.9636, respectively. The relative content of the planar 3-membered rings in double pulse irradiated zone is higher than that in single pulse irradiated zone, indicating a stronger modification and higher each rate induced by fs laser double pulses.

Further experiments were carried out to probe the internal structural change in the materials exposed by fs laser double pulses with various pulse delays. Each site is irradiated by 500 shots, with a fixed total laser fluence of 9.46 J/cm^2^. Figure 6(b) shows the percent area of the total reduced spectrum under D_2_ line of different pulse delays varying from 0 to 2000 fs. Consistent with our experimental results of etch rate enhancement shown in Fig. 3, there exists a fluctuation with the increase in the pulse delay. The strongest D_2_ peak intensity appears at a pulse delay of 350 fs.

The fundamental reason of the differences in modification induced by single and double pulses is the localized control of transient electrons dynamics by temporal shaping of fs laser pulses. During fs laser irradiation, the free electron density can be increased by several to ten orders of magnitude. Such a huge change in the free electron density also induces great changes in localized transient material properties. In the area being irradiated by fs pulses, the reflectivity of the dielectric material significantly changes and tremendously reshapes the ultrafast laser field, which strongly affects the photon energy absorption. The internal structural modification of the materials depends on the photon absorption efficiency^17^. Using the plasma model developed by us^18^, the free electron generation can be calculated by the following expression, in which the electron decay term is also considered:


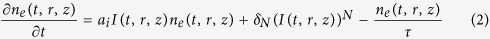


where *t* is the time; *r* is the distance to the Gaussian beam axis; *z* is the depth from the surface of the bulk material; *τ* is the decay time constant; *n*_*e*_*(t,* *r,* *z)* is the free electron density; *a*_*i*_ is the avalanche ionization constant; *I* (*t,* *r,* *z*) is the laser intensity inside the bulk material; and *δ*_*N*_ is the cross section of *N*-photon absorption.

The plasma excited by the first subpulse strongly reshapes the original laser beam, which can be quantified by the transient reflectivity during the pulse irradiation. Thus the laser intensity distribution is expressed as^12^,^18^:


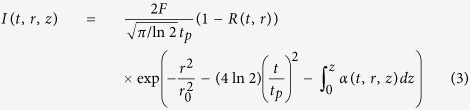


where *F* is the laser fluence; *t*_*p*_ is the pulse duration; *R* (*t,* *r*) is the reflectivity; *r*_0_ is the waist radius of the laser beam; and *α* (*t,* *r,* *z*) is the absorption coefficient.

**Figure 7:**
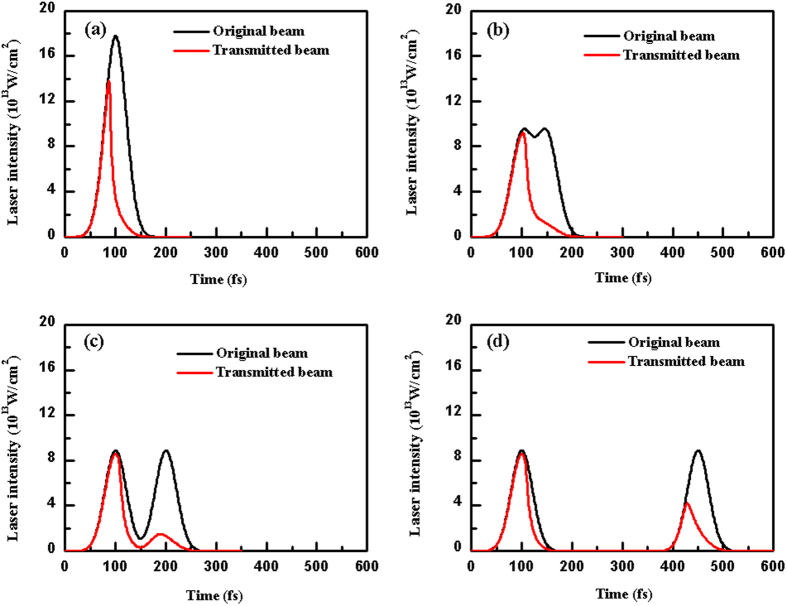
Center laser intensity distributions at different pulse delays: (**a**) 0, (**b**) 50, (**c**) 100, and (**d**) 350 fs (the laser fluence is fixed at 9.46 J/cm^2^ and the energy distribution ratio is 1:1).

From the calculation, when the pulse delay is shorter than 350 fs, the laser intensity transmitted into the material is lower than that of the original beam. With the increase in the pulse delay, the effect of the laser beam reshape tends to be weaker; and the transmitted energy obviously increases, as shown in Fig. 7. It contributes to the enhancement of the photon absorption efficiency and result in the modification improvement in the irradiated zone. Due to the modification induced by the pulse train, more 3- and 4-membered ring structured Si−O bond are formed, which have greater susceptibility to the HF etching than the stable 6-membered ring structure in the pristine fused silica^19^. Thus, the irradiated areas have an increased solubility to acids, and the chemical reactivity in reactions with HF improves. However, when the pulse delay continues to be prolonged (>350 fs), the electron decay term begins to play a dominant role^20^. In this case, free electrons excited by the first subpulse decay and even diminish before being exposed by the second subpulse. As a result, the photon absorption efficiency decreases, leading to the overall decrease in the etch rate.

**Figure 8:**
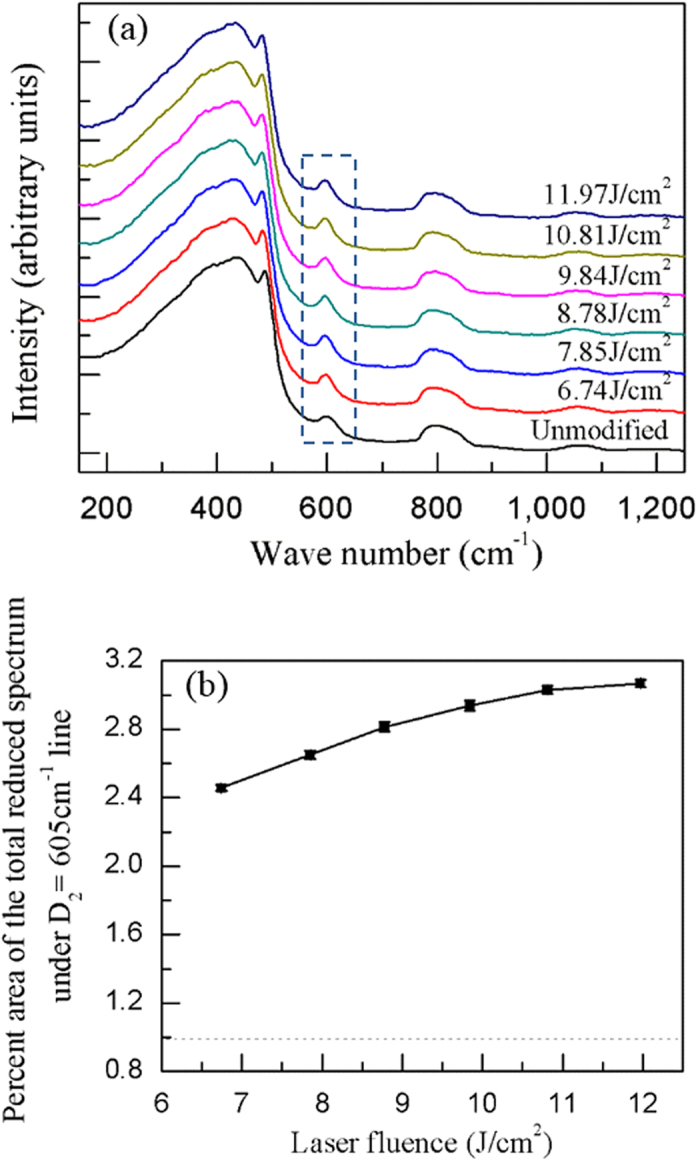
(**a**) The normalized Raman spectra of modified regions irradiated by fs laser double pulse with different laser fluences. (**b**) The percent area of the total reduced Raman spectrum under the D_2_ line versus the laser fluence, where the dashed line represents that of the unmodified original fused silica. The pulse delay is fixed at 350 fs and each site is irradiated by 500 shots. The measurements are conducted in a regions located 5 μm below the surface.

Besides, the increase in the etch rate with the increasing laser fluence could also be explained by the micro-Raman spectra. Figure 8(a) shows the normalized Raman spectra of the original fused silica and the modified zones irradiated by fs double pulses of different laser fluences. The D_2_ peak intensity increases obviously after the irradiation by the fs laser, compared with the original material. To quantitatively study the change in the D_2_ peak, we plotted the percent area under D_2_ line as a function of the laser fluence. As shown in Fig. 8(b), the D_2_ peak area increases with the increasing laser fluence before reaches a saturation. This trend agrees with the variation of etch rate in our experiments.

In conclusion, an efficient, flexible method for large-area manufacturing of concave microarrays on fused silica substrates has been developed by enhanced chemical etching using fs laser pulse trains. Compared with the conventional single pulses, the etch rate is enhanced by a factor of 37 times using the pulse shaping technology. Moreover, the shape of the micropits is tunable by designing the processing parameters, such as the fs laser pulse delay, laser fluence, and pulse energy distribution ratio of the pulse train. The effective control of localized transient free electron densities by temporally shaping fs laser pulses contributes to the etch rate enhancement and better controllability in the laser irradiated zone. This work has the potential to provide new insight into the highly efficient and controllable fabrication of microstructures in fused silica.

## Methods

### Femtosecond laser pulse irradiation

A Spectra Physics Spitfire regenerative amplifier (800 nm, 50 fs) was used with a repetition rate of up to 1 kHz and a pulse energy of up to 3 mJ. The irradiation time (number of pulse bursts) was precisely controlled by an electromechanical shutter. By combining a half-wave plate with a polarizer, the energy of the laser pulses was continuously varied. The fs laser pulse was temporally shaped to a pulse train with a certain pulse delay and energy distribution ratio between the subpulses by a pulse shaper (BSI MIIPS BOX 640), the principle of which is explained in detail by Weiner^21^. The pulses were directed into a 20× microscope objective (N.A. = 0.45) and focused at a normal incidence onto the sample mounted on a six-axis motion stage (M-840.5DG, PI, Inc.) with positioning resolution of 1 μm. The samples were 1.0 mm thick, double-sided polished fused silica glass.

### Chemical etching

After femtosecond laser irradiation, the samples were treated in ~8% HF aqueous solution assisted by an ultrasonic bath. The chemical etching was enhanced in the laser-modified areas, generating concave spherical structures. Finally, the samples were cleaned by an ultrasonic bath in acetone, alcohol, and deionized water for 10 minutes, respectively, and then dried in ambient air.

### Characterization

SEM and AFM were used to characterize the morphology of the concave microstructures on the fused silica substrate. Micro-Raman spectroscopy, excited by a 488-nm laser beam, was conducted to elucidate the internal structural change in the fused silica after fs laser irradiation.

## Additional Information

**How to cite this article**: Zhao, M. *et al.* Controllable high-throughput high-quality femtosecond laser-enhanced chemical etching by temporal pulse shaping based on electron density control. *Sci. Rep.* **5**, 13202; doi: 10.1038/srep13202 (2015).
